# Medical Knowledge

**Published:** 1856-10

**Authors:** J. R. Freese

**Affiliations:** Bloomington, Ill.


ARTICLE V.—*Medical Knowledge.* By J. R. FREESE, M.D., of Bloomington, Ill.

From the days of Hippocrates, down almost to the present, Medical knowledge has been confined to the few, while the great mass of mankind have neither sought or cared to know anything concerning it.

Twenty years ago, the idea of having physiology taught in our common schools would have been thought preposterous; and even the leading colleges of our country, while they were careful to teach their classes Xenophon's Anabasis and Memorabilia, Demosthenes de Corona, Evidences of Christianity, Natural Philosophy, Aristotle's Art of Poetry, Geology, Zoology, &c., yet they never once thought it important to add the study of ONE'S SELF; as though to *know ourselves* were of but little importance in comparison with knowing the "dead languages," and sciences, from which we could never hope to reap any practical advantage!

I, for one, am exceedingly glad that a change is beginning to take place on this all-important subject; that the first principles of our noble science are beginning to be disseminated among the people; that our common schools have introduced the study of physiology, and our literary colleges made it a part of their studies; that well-informed physicians have thought it no disgrace to our profession, and no breach of medical ethics to lecture to the people on physiology and hygienic law; that physicians, instead of holding themselves aloof from the people, as though they were part and parcel of a separate creation, are beginning to mix with the people and converse with them in such a plain yet scientific manner as to enlighten, instead of be-fogging the minds of their patrons. To me these signs are propitious, and I hail them with great joy.

I am also well pleased to notice a greater amount of sociability among the members of our own profession; and a disposition not only to permit and urge on the spread of true medical knowledge among the people, but a free interchange of sentiment, to spread knowledge among ourselves. Dr. Palmer, of Chicago, in his report on the "Organization of

State and County Societies," made to the American Medical Association, at its recent meeting at Detroit, has done the profession a great service; and, if his plan is adopted and carried out, not only will the members of the profession be benefited, but the people of every State and County, in which such an organization may be effected, will be correspondingly benefited.

In proportion to the amount of medical knowledge among the people, will be the respect and honor paid to the well-informed physician. Quackery can only flourish among the ignorant — and the greater the ignorance, the greater the quackery. A competent physician has no need of cloaks or seals to his profession. He knows his business, and if he is so fortunate as to have a set of patrons who understand something of the human system, of the laws of health, and the power of medicines, he will grow up among them to be loved, honored, and cherished for his knowledge of head, and goodness of heart.

Of course I admit, that the *amount* of medical knowledge necessary to make a competent physician and surgeon, must always be confined to the few, comparatively, because there are only a few that will go through the long study, and spend the amount of time and money, necessary to such an end: yet, in admitting this, it lessens not my hope and my strong desire that true medical knowledge will continue to spread among the people; that it will continue to be made a part of common school and collegiate education; that the professors of our medical colleges, and especially our medical journals, will mingle more with, and be scattered more among the people—teaching them a knowledge of themselves, and the means whereby to prevent and to cure the many ills to which human flesh is heir to.

I look forward to the effects of such a spreading of medical knowledge as a kind of medical millenium; and, having strong faith in its steady and rapid approach, I hope, myself, to live long enough to see it and enjoy a share of its professional blessings.

